# Citywide Integrated *Aedes aegypti* Mosquito Surveillance as Early Warning System for Arbovirus Transmission, Brazil

**DOI:** 10.3201/eid2804.211547

**Published:** 2022-04

**Authors:** André S. Leandro, Wagner A. Chiba de Castro, Renata D. Lopes, Robson M. Delai, Daniel A.M. Villela, Rafael Maciel de-Freitas

**Affiliations:** Secretaria Municipal de Saúde de Foz do Iguaçu, Foz do Iguaçu, Brazil (A.S. Leandro, R.D. Lopes);; Instituto Oswaldo Cruz, Rio de Janeiro, Brazil (A.S. Leandro, R. Maciel-de-Freitas);; Universidade Federal Latino-Americana, Foz do Iguaçu (W.A. Chiba de Castro, R.D. Lopes);; Centro de Medicina Tropical da Tríplice Fronteira, Foz do Iguaçu (R.M. Delai);; Programa de Computação Científica, Fiocruz, Rio de Janeiro (D.A.M. Villela); Bernhard Nocht Institute for Tropical Medicine, Hamburg, Germany (R. Maciel-de-Freitas)

**Keywords:** vector-borne infections, viruses, zoonoses, mosquitoes, dengue, Zika, chikungunya, entomologic surveillance, vector control, vectorial capacity, disease transmission, epidemiology, arbovirus, Brazil, Aedes aegypti

## Abstract

Arbovirus epidemiology lacks efficient and timely surveillance systems with accurate outbreak alert signals. We devised a citywide integrated surveillance system combining entomologic, epidemiologic, and entomo-virologic data gathered during 2017–2020 in Foz do Iguaçu, Brazil. We installed 3,476 adult mosquito traps across the city and inspected traps every 2 months. We compared 5 entomologic indices: traditional house and Breteau indices for larval surveys and trap positivity, adult density, and mosquitoes per inhabitant indices for adult trapping. We screened for dengue, Zika, and chikungunya viruses in live adult *Aedes aegypti* mosquitoes collected from traps. Indices based on adult mosquito sampling had higher outbreak predictive values than larval indices, and we were able to build choropleth maps of infestation levels <36 h after each round of trap inspection. Locating naturally infected vectors provides a timely support tool for local public health managers to prioritize areas for intervention response to prevent virus outbreaks.

## Abstract 2

Infestation indices based on adult trapping predicted dengue outbreaks better than larval indices did.

The *Aedes aegypti* mosquito is the primary vector of arboviruses such as dengue (DENV), Zika (ZIKV), chikungunya (CHIKV), and yellow fever. This mosquito species is common in urbanized areas in the tropics because it is highly adapted to live in close association with humans, preferentially feeding on blood of human hosts and laying eggs in containers located around human dwellings (*1*–*6*). Estimates indicate that ≈3 billion persons live in areas with ongoing DENV transmission (*7*).

Traditional entomologic surveillance for *Ae. aegypti* mosquitoes is based on periodic inspections of larvae and pupae in domestic breeding sites, which provide measures of infestation known as the house index (HI), the percentage of houses in which >1 larvae or pupae was collected, and Breteau index (BI), the number of containers positive for larvae or pupae divided by the number of inspected houses. By using available infestation data, public health managers intensify control strategies in the areas with higher indices. Of note, indices based on collection of immature mosquitoes face many criticisms because surveys are costly to perform with the frequency required for adequate surveillance; indices are highly dependent on the agent’s motivation to effectively search for larvae in myriad container types, including cryptic and hard-to-access containers; surveys do not consider container productivity (i.e., these surveys might only provide measures of presence or absence immature mosquitoes); and larval density has proven to be a poor indicator of adult mosquito density (*8*–*12*).

Traps capturing adult mosquitoes could be a promising alternative to larval surveys because they sample the vector life stage that is directly responsible for transmission and provide qualitative (percent positive traps) and quantitative (number of captured mosquitoes per trap) indices (*8*,*13**–**17*). Adult traps provide relative measurements of the vector population, expressed in units of mosquitoes by area, mosquitoes per person, or mosquitoes per trap (*8*,*18*,*19*). Therefore, adopting of adult traps in an arbovirus-endemic setting likely would provide relevant information regarding the spatiotemporal dynamics of *Ae. aegypti* mosquitoes.

Effective arbovirus surveillance should be able to accurately predict when and where an outbreak will occur. Routine virologic surveillance in field-caught *Ae. aegypti* mosquitoes, (entomo-virologic surveillance) is one measure that could be adopted to enhance surveillance effectiveness (*20*). In DENV-endemic settings of developing countries, screening for natural infection in field-caught *Ae. aegypti* mosquitoes has been performed in various situations but rarely as a component of a long-term routine surveillance to direct control interventions to critical areas (*21*–*23*). By adding entomo-virologic surveillance to routine surveillance based on large-scale adult mosquito trapping across an entire city, health managers ideally would be able to identify hotspots of disease transmission and intensify vector control in those regions before human cases arose (*19*,*24*).

We report on a 4-year integrated citywide vector surveillance approach that involved extensive use of adult mosquito traps, molecular diagnostic testing for natural arbovirus infection in live collected mosquito specimens, construction of transmission risk maps, and performance of timely vector control intervention <48 h after mosquito collection. In this scheme, vector control was intensified in areas with higher risk for transmission instead of maintaining homogeneous vector control efforts over the landscape. In addition, we evaluated the correspondence of larval- and adult-based indices with the epidemiologic trend in the city of Foz do Iguaçu, Brazil, during 2017–2020.

## Methods

### Study Site

We implemented an entomo-virologic surveillance system in the city of Foz do Iguaçu (25°30′58″S, 54°35′07″W), Brazil, which is located on the triple border with Argentina and Paraguay. Foz do Iguaçu has ≈250,000 inhabitants and an intense daily population movement across the 3 countries’ border cities. Foz do Iguaçu is divided into 73 urban areas of ≈1,500 premises each (*25*), plus 3 rural areas that were not included in this study. We defined premises as a property occupied by a residence or a business at ground level. According to the Brazil Ministry of Health, apartment buildings are not included, and surveillance and vector control interventions take place only at the foyer. The climate in Foz do Iguaçu is classified as humid tropical, according to the Köppen-Geiger system, and is characterized by hot and humid summers (mean temperature >27°C) and cold to mild winters (mean temperature <15°C), with an annual rainfall >1,850 mm.

### Adult Mosquito Collection

During January 2017–December 2020, a total of 3,476 Adultraps (Berdon, https://adultrap.com.br) were installed in the city, and 1 trap could be found in the peridomestic environment for every 25 premises. This system was originally designed to capture gravid *Ae. aegypti* female mosquitoes during oviposition because Adultraps use water as the principal attractant. These traps have an opening on the top where females enter, then are trapped in an interior chamber (*16*,*26*). Water remains confined in a compartment at the bottom of the trap that the mosquitoes cannot access, thus deterring egg laying. Local health agents visit all Adultraps every 2 months, within the first 4 days of the first week of odd months, when agents usually conduct larval surveys as part of traditional entomologic index. Therefore, during the study period, the 3,476 Adultraps were inspected 24 times in the same premises, a total of 83,424 trap inspections.

### Entomologic Indices

Besides the traditional HI and BI based on larval surveys, the Adultrap inspections produced 3 entomologic indices based on adult collections. The trap positivity index (TPI) is the number of positive traps among the total number of traps inspected multiplied by 100; the adult density index (ADI) is the total number of *Ae. aegypti* mosquitoes captured divided by the total number of inspected traps multiplied by 100; and the mosquitoes per inhabitant index (MII) is the total number of adult *Ae. aegypti* mosquitoes collected, divided by the number of persons in each house with an Adultrap multiplied by 1,000. We calculated all entomologic indices every 2 months during 2017–2020, a total of 24 observations per index.

### Entomo-Virologic Screening

Mosquitoes collected alive during each 2-month period were sent to the entomology laboratory for further taxonomic identification by using appropriate keys. Mosquitoes classified as *Ae. aegypti* were placed in cryogenic tubes for diagnosis of arbovirus infection by quantitative real-time PCR (qPCR). Depending on the number of mosquitoes captured in traps on the same city block, we pooled <10 mosquitoes per block, separating male from female mosquitoes. We calculated minimum infection rate (MIR) by dividing the number of positive pools by the total specimens tested, then multiplied by 1,000 (*27*). To estimate MIR, we used only data from DENV-positive pools because only a few pools were positive for ZIKV or CHIKV.

### RNA Extraction and Real-Time qPCR

We extracted viral RNA from *Ae. aegypti* mosquitoes by using the MagMAX Viral/Pathogen Nucleic Acid Ultra Isolation KIT (Applied Biosystems/Thermo Fisher Scientific, https://www.thermofisher.com), according to the manufacturer’s instructions. We added single or pooled mosquitoes to electromagnetic mixing beads (MagMAX Viral/Pathogen Binding Beads; Applied Biosystems) and macerated by using TissueLyser II (QIAGEN, https://www.qiagen.com). After RNA extraction, we separated an aliquot of 2 μL from each sample and used this to read the concentration of viral RNA recovered in a NanoDrop One^C^ Spectrophotometer (Thermo Fisher Scientific).

For arboviral genome amplification, we used the ZDC Biomol Kit (Instituto Biologia Molecular do Paraná [IBMP], https://www.ibmp.org.br) (*28*–*31*), which enables identification of ZIKV, CHIKV, and differentiation of DENV serotypes with an internal control (IC) of the reaction that uses probes specific to each molecular target. We used a 96-well QuantStudio 7 Flex Real-Time PCR System (Applied Biosystems) for PCR and analyzed results by using QuantStudioDesign and Analysis Software versions 1.3.1 and 1.5.1 (Applied Biosystems). We considered samples positive when the amplification plot curve exceeded the specific threshold for each target <35 cycle threshold.

### Epidemiologic Surveillance and Case Report

The health system in Foz do Iguaçu is composed of 30 basic health units, including 2 emergency care units, 3 private hospitals, and 1 public hospital. The Ministry of Health lists dengue, Zika, and chikungunya as diseases of compulsory notification that can be registered in any of the local health facilities. Epidemiologic surveillance for arboviruses is carried out passively after symptomatic persons seek care in the city health system. Zika and chikungunya cases were reported in the city before 2017, but no further large outbreaks were reported in Foz do Iguaçu. Thus, we restricted our analysis to suspected dengue cases reported during 2017–2020 (*25*). A suspected dengue case was reported whenever any person residing in Foz do Iguaçu received a clinical diagnosis of >1 compatible dengue symptom, including fever, headache, myalgia, arthralgia, rash, nausea, retro-orbital pain, petechiae, or malaise, in the previous 14 days. Among suspected dengue cases, 33.2% were confirmed through laboratory diagnosis.

### Geographic Information and Choropleth Maps

We recorded and stored all entomologic and epidemiologic field-derived data, such as the location and trapping history of the 3,476 adult mosquito traps, along with geocoded residential address of suspected dengue patients, in a single database (*25*). We used PostgreSQL version 9.5.7 (instaclustr, https://www.instaclustr.com) for data storage and Quantum GIS version 3.10.2 (QGIS, https://www.qgis.org) to produce maps. We used geoprocessed information and Power BI version 2.85.985.0 (Microsoft, https://powerbi.microsoft.com) to generate reports, graphs, and maps.

We created choropleth maps to help visualize the *Ae. aegypti* mosquito population among the 73 areas of the city. We built the choropleth maps in accordance with guidelines provided by the Brazil Ministry of Health, which classifies HI <1.0 as a low risk for dengue transmission, HI from 1.1–4.0 as a moderate risk, and HI >4.0 a high risk.

### Statistical Analysis

We evaluated the predictive ability of the entomologic indices by using 5 scenarios comprising comparison of dengue incidence (notifications per 100,000 inhabitants) in the same week and in 2, 4, 6, and 8 weeks after the entomologic surveys. We assessed each scenario in each index by using generalized linear mixed models (GLMM) with temporal pseudoreplication (*32*). In these models, we included HI, BI, TPI, ADI, and MII as the fixed effect in the explanatory variable indices of larvae and adult mosquitoes, and the incidence of dengue as the response variable. The continuous random-effect structure included each resampling date with 23 levels in each of the 73 areas of Foz do Iguaçu (*33*). We assumed a Gaussian distribution for the continuous response variable and implemented the models in the lme4 package in R (R Foundation for Statistical Computing, https://www.r-project.org). We obtained significance values for fixed effects in the ImerTest software package (R Foundation for Statistical Computing). We chose the best scenario by using Akaike information criteria (AIC) to rank models (ΔAICc), and calculated Akaike weights (wAICc) to evaluate the relative support of each model (*34*). We used ΔAICc to evaluate the differences in AIC score between the best model and the other models. We used Akaike weights to evaluate model selection uncertainty, which quantified the probability that the model was the best among those considered based on the data (*34*,*35*). We selected the best supported model based on rejection of GLMM null hypothesis (p<0.05), the lower AIC value, and an AIC weight >0.7 (70% confidence set) (*34*). We considered models with ΔAICc of <4.0 to have no differences (*36*). We implemented the ΔAICc and wAICc in the bbmle package (R Foundation for Statistical Computing).

## Results

### Entomologic Survey of Larvae

In each 2-month period, an average of ≈4,883 (range 4,781–5,021) premises were inspected, which correspond to ≈6.25% of houses in Foz do Iguaçu. During 2017–2020, a range of 3.5%–17.7% of inspected Adultraps were positive for mosquitoes, and an average of 9.5% of traps had >1 *Ae. aegypti* mosquito. We used the number of positive houses to create HI and the number of containers to create BI and observed strong seasonal variation; values were 7 times higher during the wet summer (November–March) than in the dry winter (July–September) (Appendix 1). The average HI of Foz do Iguaçu was 2.58% during the 24 observations of 2017–2020, and only twice was HI above the 4% alert level adopted by Ministry of Health, reaching 5.41% in March 2019 and 5.29% in May 2019.

**Figure 1:**
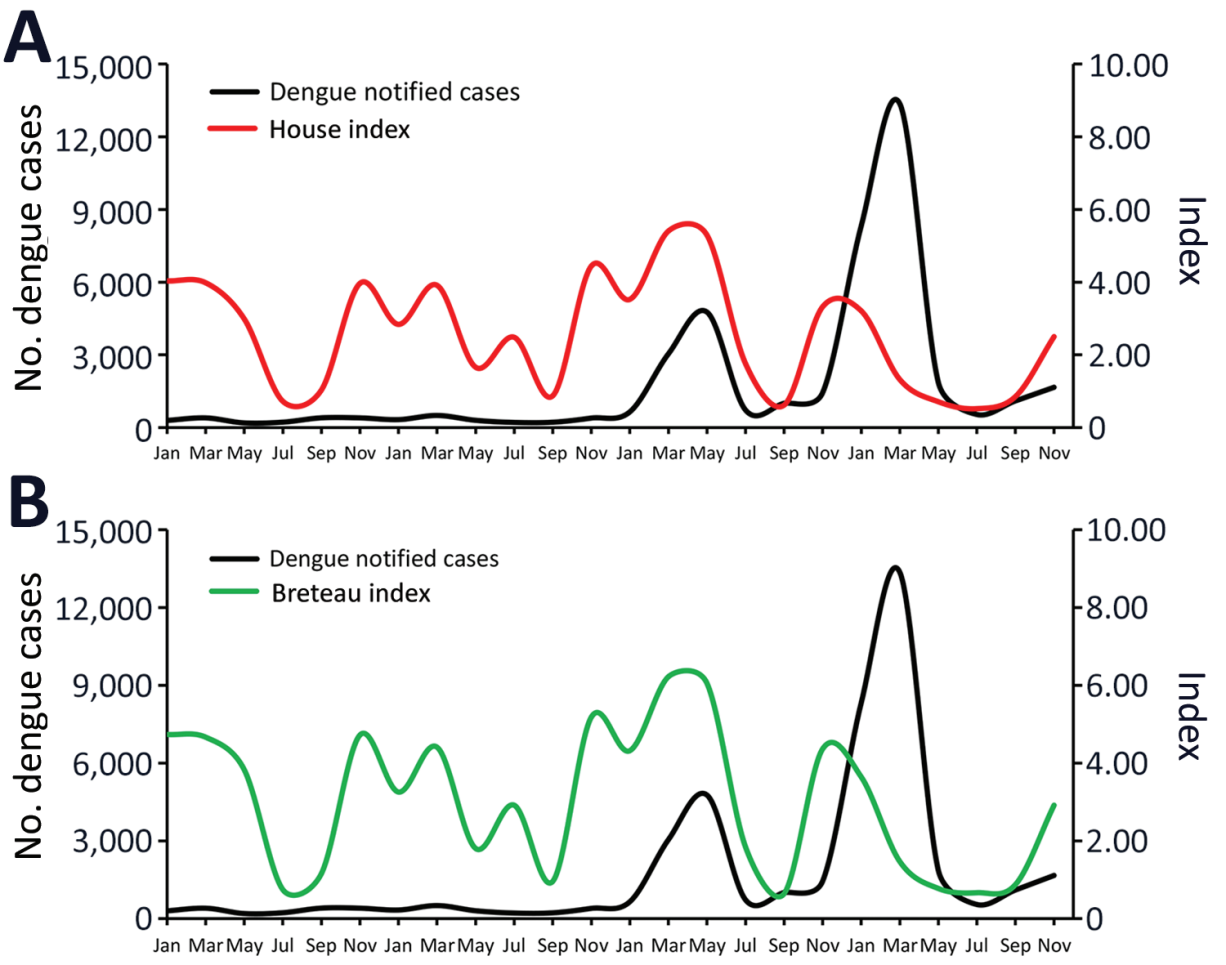
Bimonthly variation on traditional *Aedes aegypti* mosquito infestation indices based on larval surveys compared with number of reported dengue cases, Foz do Iguaçu, Brazil, 2017–2020. A) House index; B) Breteau index.

We used the number of positive houses to estimate HI and number of positive breeding sites in each of larval survey to estimate traditional BI (Figure 1). Indices based on larval surveys showed an expected seasonal variation with higher values during the rainy summer (≈November–March), but HI and BI fluctuations were only partially in accordance with the dengue notification curve (Figure 1).

### Entomologic Survey for Adult Mosquitoes

Adult *Aedes aegypti* mosquitoes were collected on the same premises where larval surveys were performed. The average number of inspected traps was 2,468 (range 2,239–2,767). Therefore, a mean of 73% of adult traps were inspected bimonthly. A total of 11,962 adult *Ae. aegypti* mosquitoes were captured in the adult traps, showing a massive predominance of female mosquitoes, 95.4% of all captured insects (Appendix 1).

**Figure 2:**
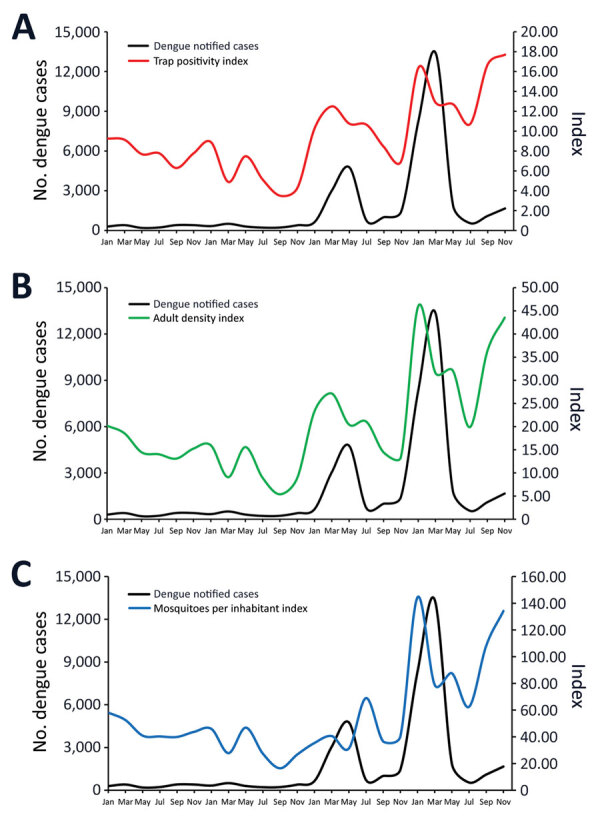
Bimonthly variation on *Aedes aegypti* infestation indices based on surveys of adult mosquitoes captured compared with number of reported dengue cases, Foz do Iguaçu, Brazil, 2017–2020. A) Trap positivity index; B) adult density index; C) mosquitoes per inhabitant index.

In contrast to the indices based on larval surveys, indices based on adult capturing corresponded more closely to the dengue notification curve (Figure 2). Ultimately, we observed high infestation levels based on adult indices in Foz do Iguaçu that aligned with dengue notification.

### Entomo-Virologic Survey

Of the 11,962 adult *Ae. aegypti* mosquitoes trapped during 2017–2020, a total of 1,563 (13.1%) were captured alive. In addition, 1,459 (93.3%) were screened for arbovirus infection through real-time qPCR. Subsequently, mosquitoes were screened for infection in 20/24 months of thorough monitoring that summed up 221 pools (Table). From the 221 pools tested, 29 (13.1%) were positive for arboviruses, among which 22 (75.9%) pools were positive for DENV, 3 (10.3%) for ZIKV, and 4 (13.8%) for CHIKV. The average MIR for DENV was 42.6 (range 19.6–75.0), and MIR peaked in March 2020. The entomo-virologic results of natural DENV, ZIKV, and CHIKV infection in field-caught mosquitoes was available <36 hours after Adultrap inspection.

**Table:** Akaike information criteria results ranking the most parsimonious a priori models (descending order) that predict the incidence of dengue cases based on surveys of *Aedes aegypti* mosquitoes, Foz do Iguaçu, Brazil*

Scenarios	Index	ΔAICc	wAICc
After 4 weeks	Mosquitoes per inhabitant	0.0	0.6079
	Adult density	0.9	0.3908
	Trap positivity	12.3	0.0013
After 2 weeks	Adult density	406.0	<0.001
	Trap positivity	407.8	<0.001
	Mosquitoes per inhabitant	407.8	<0.001
	Breteau	426.4	<0.001
After 6 weeks	Mosquitoes per inhabitant	1,151.2	<0.001
	Adult density	1,156.3	<0.001
	Trap positivity	1,177.7	<0.001
During the same week	Adult density	2,146.8	<0.001
	Mosquitoes per inhabitant	2,148.0	<0.001
	Trap positivity	2,151.3	<0.001
	House	2,159.7	<0.001
	Breteau	2,161.1	<0.001
After 8 weeks	Adult density	2,286.3	<0.001
	Mosquitoes per inhabitant	2,288.5	<0.001
	Trap positivity	2,297.6	<0.001

*Based on p≤0.05 in a generalized linear mixed model. Each model was denoted as a specific scenario, from the same week to 8 weeks after entomologic surveys, and index. House and Breteau indices are based on larval surveys of *Aedes aegypti* mosquitoes; mosquito per inhabitant, trap positivity, and adult density indices are based on adult trapping. ΔAICc, difference in corrected Akaiks information criteria; wAICc, weights of corrected Akaike information criteria.

### Dengue Prediction of Entomologic Indices

All entomologic indices based on adult sampling (TPI, ADI, and MII) showed a statistically significant relationship with dengue incidence in Foz do Iguaçu during 2017–2020 (Table). Indices based on larval surveys had limited statistically significant relationships with dengue incidence in the same week for both BI and HI and for BI after 2 weeks. Of note, indices based on adult trapping best predicted the incidence of dengue after 4 weeks, with emphasis on ADI and MII (Table). Adult indices showed a stronger prediction of future dengue incidence than traditional larval surveys indices on the basis of GLMM results for each index in the 5 scenarios (Appendix 2 Tables 1–5). Of note, coefficients for HI and BI were negative in most scenarios whereas positive coefficients were observed for TPI, ADI, and MII.

### Choropleth Maps

**Figure 3:**
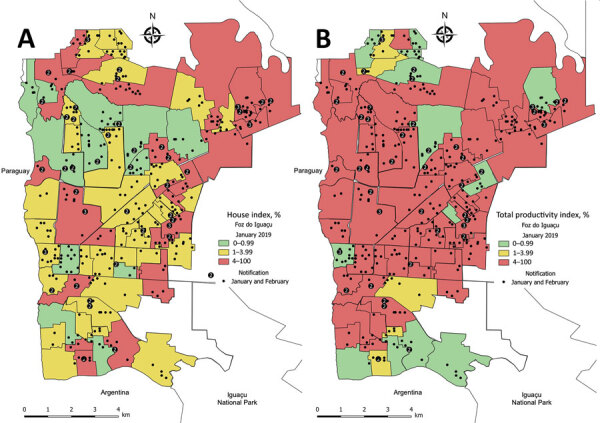
Choropleth maps comparing larval and adult *Aedes aegypti* mosquito infestation indices and dengue notifications in 73 urban areas of Foz do Iguaçu, Brazil, January–February 2019. A) Traditional house index (HI) calculated from larval surveys; B) trap positivity index (TPI) calculated from dengue virus positivity among captured adult *Ae. aegypti* mosquitoes. Dots represent dengue notification and numbers inside dots represent the total of dengue cases reported on that city block.

We constructed maps for the 5 entomologic indices in each of the 24 months of collection during 2017–2020. We selected January 2019 to illustrate the differences between maps based on larval surveys (HI) from the one using adult trapping (TPI), and considered HI and TPI analogous (Figure 3). Of note, January 2019 marked the initial rise in dengue cases in the city, which peaked during March–May 2019, the moment in which a sensitive tool could foresee an increase in dengue transmission. In January 2019, HI classified 52 (71.2%) areas as being low and moderate risk for dengue transmission, whereas TPI estimated 25 (34.2%) areas under the same risk. The relative frequency of high-risk areas before the start of a dengue outbreak increased from 28.7% when measured by HI to 65.8% when measured by TPI (Figure 3).

## Discussion

Epidemiologic surveillance is defined as the systematic collection, analysis, and interpretation of determinants of disease activity to support the planning and implementation of further actions to mitigate disease burden. Systematic literature reviews have stressed a general lack of evidence for the usefulness of arboviral surveillance for early outbreak detection and emphasized the lack of indicators and alert signals to trigger response (*37*,*38*). When most arbovirus-endemic countries rely on passive surveillance with clinical but few laboratory diagnostics to confirm infection, epidemiologic data frequently are not able to provide a sensitive alert signal before an outbreak takes place. We report on implementation of a citywide integrated surveillance system using entomologic, epidemiologic, and entomo-virologic data gathered during a 4-year period. The extensive fieldwork provided a large dataset and enabled robust analysis. The entomologic indices based on adult trapping provided a more reliable alert signal of dengue outbreaks than widespread traditional indices based on larval surveys.

Dengue entomologic surveillance using larval inspections is a time- and resource-consuming activity widely used in many tropical countries (*8*,*10*,*39**–**42*). Larval surveys can identify key containers but often fail to provide fast or localized measurements of mosquito abundance. In this context, adopting adult mosquito traps as a complementary approach can improve dengue vector surveillance by providing information previously unknown in larval surveys, such as adult female mosquito abundance (*8*,*43*,*44*). Traditional larval surveillance indices often fail to demonstrate a strong correlation with adult mosquito density and dengue transmission. Thus, developing other indices to serve as indicators of an imminent dengue outbreak should be encouraged (*41*). Of note, TPI, ADI, and MII were developed by using Adultrap and thus should not be seen as universal adult indices. Although specific for Adultrap, our results highlight that analogous approaches, such as extensive time series data, traditional entomologic data, and high cover in a city, should be pursued by using other traps (*41*).

Ultimately, even though 73 areas of the city were tested, the HI rarely reached values above the 4% alert threshold; the only exceptions were in March 2019 (5.41%) and May 2019 (5.29%). Furthermore, the most intense dengue transmission peak in Foz do Iguaçu was recorded during January–May 2020. In this window, we had 3 HI estimates, 3.21 in January, 1.32 in March, and 0.7 in May. We observed the same pattern of poor correlation with local dengue transmission for BI, evincing criticisms directed to larval surveys as both entomologic and epidemiologic indicators. Instead, indices based on adult trapping showed low variation during the nonendemic years of 2017–2018 and peaked accordingly in the 2019 and 2020 dengue seasons.

Standard larval surveys were not sufficient to issue proper alerts in Foz do Iguaçu. By comparison, Adultraps detected increased mosquito infestation during the dengue transmission seasons, indicating the system’s ability to detect mosquito density variation and thus the likelihood of generating indices that could be used as part of an early warning system to trigger vector control response. The greater sensitivity of traps to mosquito density variation is probably because they can cover >1 premises, whereas surveys of immature mosquitoes only encompass those houses included in the sample (*8*).

Comparing the predictive ability of traditional versus adult indices revealed that indices based on adult trapping consistently performed much better than indices based on larval surveys. In fact, we observed negative GLMM coefficients for HI and BI but saw positive estimates for indices based on adult trapping. In addition, MII, ADI, and TPI performed better as predictive indicators of dengue outbreaks 4 weeks after the trapping period. Therefore, local health managers would have ≈1 month after estimating the index values to promote and intensify vector control in areas with higher risk on a choropleth map. In addition, health managers could create additional criteria to prioritize areas for vector control in case the cost to cover all high-risk areas of a city becomes too expensive to be covered by health agencies.

One criterion that could be used to prioritize areas is the occurrence of *Ae. aegypti* female mosquitoes naturally infected with DENV, ZIKV, or CHIKV. In Foz do Iguaçu, inspection of the 3,476 Adultraps took 4 days, and real-time qPCR results were available, on average, 36 h after all Adultraps were inspected and live mosquitoes collected. Thus, within 5 days of starting trap inspection, additional entomologic information, such as geographic position of traps, the infestation index, and the choropleth maps, were made available for local health managers. In the early hours of the next business day, the local health manager could meet with field supervisors to decide which area to prioritize and which vector control activities to perform considering local contexts (*45*,*46*). Therefore, a week after the start of Adultrap inspection, the dengue transmission risk among the 73 areas of Foz do Iguaçu would be known by the local health managers, triggering vector control interventions in prioritized areas.

In conclusion, traditional entomologic indices have shown a poor relationship with dengue transmission, if any (*47**–**49*). We conducted a 4-year citywide study to deepen the entomologic and epidemiologic features of dengue transmission in Foz do Iguaçu by focusing on developing indicators based on adult mosquito trapping. We demonstrated the process we used to develop the 3 adult trapping indices, all of which have a higher prediction behavior to foresee dengue outbreaks than the widely adopted traditional larval survey indices. Our proposed surveillance system can predict a dengue outbreak with high accuracy, and indices based on adult trapping are able to predict a dengue outbreak 4 weeks after DENV detection in adult mosquitoes. In addition, adoption of easily accessible technological resources makes it possible for the model to be replicated to other localities.

Appendix 1 Summary of entomologic, epidemiologic, and entomo-virologic data gathered during citywide arbovirus surveillance, Foz do Iguaçu, Brazil, 2017–2020.

Appendix 2 Additional information on models assessing the predictions of various indices among *Aedes aegypti* mosquitoes on the incidence of dengue, Foz do Iguaçu, Brazil, 2017–2020.
