# Supplementary material for: Citywide Integrated Aedes aegypti Mosquito Surveillance as Early Warning System for Arbovirus Transmission, Brazil
Source: Emerg Infect Dis. 2022 Apr;28(4):707–16. doi: 10.3201/eid2804.211547 (PMC8962889; doi:10.3201/eid2804.211547)
Supplement: Appendix 2 — Additional information on models assessing the predictions of various indices among Aedes aegypti mosquitoes on the incidence of dengue, Foz do Iguaçu, Brazil, 2017–2020. [file 21-1547-Techapp-s2.pdf]

Article DOI: https://doi.org/10.3201/eid2804.211547

Citywide Integrated *Aedes aegypti* Mosquito Surveillance as Early Warning System for Arbovirus Transmission, Brazil

Appendix 2

Appendix 2 Table 1. Results of the generalized linear mixed models assessing the predictions of house index among *Aedes aegypti* mosquitoes on the incidence of dengue in 5 different scenarios, Foz do Iguaçu, Brazil, 2017–2020

Scenarios	Factor	Estimate	Standard error	*t* value
Same week	Intercept	91.797	6.634	13.837*
	House index	−8.355	2.766	−3.021†
After 2 weeks	Intercept	59.240	3.985	14.867*
	House index	−3.178	1.648	−1.929
After 4 weeks	Intercept	49.3625	3.5937	13.736*
	House index	−0.9941	1.4752	−0.674
After 6 weeks	Intercept	55.9449	5.0541	11.069*
	House index	0.2192	2.0948	0.105
After 8 weeks	Intercept	73.520	6.991	10.517*
	House index	3.722	2.914	1.277

*Statistically significant result, p<0.0001.
†Statistically significant result, p<0.001.

Appendix 2 Table 2. Results of the generalized linear mixed models assessing the predictions of Breteau index among *Aedes aegypti* mosquitoes on the incidence of dengue in 5 different scenarios, Foz do Iguaçu, Brazil, 2017–2020

Scenarios	Factor	Estimate	Standard error	*t* value
Same week	Intercept	90.618	6.527	13.883*
	Breteau index	−6.485	2.271	−2.856†
After 2 weeks	Intercept	59.528	3.917	15.199*
	Breteau index	−2.762	1.353	−2.041‡
After 4 weeks	Intercept	50.139	3.540	14.16*
	Breteau index	−1.455	1.212	0.23
After 6 weeks	Intercept	55.8899	4.9784	11.23*
	Breteau index	0.2926	1.7220	0.865
After 8 weeks	Intercept	73.390	6.876	10.673*
	Breteau index	3.092	2.395	0.197

*Statistically significant result, p<0.0001.
†Statistically significant result, p<0.001.
‡Statistically significant result, p<0.05.

Appendix 2 Table 3. Results of the generalized linear mixed models assessing the predictions of trap positivity index among *Aedes aegypti* mosquitoes on the incidence of dengue in 5 different scenarios, Foz do Iguaçu, Brazil, 2017–2020

Scenarios	Factor	Estimate	Standard error	*t* value
Same week	Intercept	52.0100	8.4248	6.173*
	Trap positivity index	3.1847	0.7066	4.507*
After 2 weeks	Intercept	35.7530	5.0457	7.086*
	Trap positivity index	2.1327	0.4222	5.052*
After 4 weeks	Intercept	20.1856	4.5308	4.455*
	Trap positivity index	2.8842	0.3768	7.654*
After 6 weeks	Intercept	13.4418	6.3652	2.112†
	Trap positivity index	4.3620	0.5337	8.174*
After 8 weeks	Intercept	35.2194	8.8110	3.997*
	Trap positivity index	4.7323	0.7384	6.409*

*Statistically significant result, p<0.0001.
†Statistically significant result, p<0.05.

Appendix 2 Table 4. Results of the generalized linear mixed models assessing the predictions of adult density index among *Aedes aegypti* mosquitoes on the incidence of dengue in 5 different scenarios, Foz do Iguaçu, Brazil, 2017–2020

Scenarios	Factor	Estimate	Standard error	*t* value
Same week	Intercept	56.0966	7.2512	7.736*
	Adult density index	1.3371	0.2571	5.201*
After 2 weeks	Intercept	40.8019	4.3460	9.388*
	Adult density index	0.8314	0.1536	5.411*
After 4 weeks	Intercept	25.3389	3.9251	6.456*
	Adult density index	1.1704	0.1374	8.519*
After 6 weeks	Intercept	19.2142	5.4924	3.498*
	Adult density index	1.8589	0.1943	9.568*
After 8 weeks	Intercept	42.0809	7.5616	5.565*
	Adult density index	1.9786	0.2681	7.381*

*Statistically significant result, p<0.0001.

Appendix 2 Table 5. Results of the generalized linear mixed models assessing the predictions of mosquitoes per inhabitant index for the incidence of dengue cases in 5 different scenarios, Foz do Iguaçu, Brazil, 2017–2020

Scenarios	Factor	Estimate	Standard error	*t* value
Same week	Intercept	56.4221	7.1622	7.878*
	Mosquitoes per inhabitant index	0.4333	0.0818	5.297*
After 2 weeks	Intercept	40.94	4.301	9.519*
	Mosquitoes per inhabitant index	0.2670	0.0489	5.460*
After 4 weeks	Intercept	25.34	3.886	6.522*
	Mosquitoes per inhabitant index	0.3801	0.04362	8.715*
After 6 weeks	Intercept	18.32	5.437	3.369*
	Mosquitoes per inhabitant index	0.6147	0.06159	9.979*
After 8 weeks	Intercept	33.61	7.623	4.409*
	Mosquitoes per inhabitant index	0.6743	0.08522	7.913*

*Statistically significant result, p<0.0001.
